# Citizen Science in Postsecondary Education: Current Practices and Knowledge Gaps

**DOI:** 10.1093/biosci/biab125

**Published:** 2022-01-05

**Authors:** Heather D Vance-Chalcraft, Allen H Hurlbert, Jennifer Nesbitt Styrsky, Terry A Gates, Gillian Bowser, Colleen B Hitchcock, Michelle Anne Reyes, Caren B Cooper

**Affiliations:** East Carolina University, Greenville, North Carolina, United States; Undergraduate Student Experiences with Citizen Science Research Coordination Network; University of North Carolina, Chapel Hill, Chapel Hill, North Carolina, United States; Undergraduate Student Experiences with Citizen Science Research Coordination Network; University of Lynchburg, Lynchburg, Virginia, United States; Undergraduate Student Experiences with Citizen Science Research Coordination Network; Department of Biological Sciences, North Carolina State University, Raleigh, North Carolina, United States; Undergraduate Student Experiences with Citizen Science Research Coordination Network; Colorado State University, Fort Collins, Colorado, United States; Undergraduate Student Experiences with Citizen Science Research Coordination Network; Brandeis University, Waltham, Massachusetts, United States; Undergraduate Student Experiences with Citizen Science Research Coordination Network; East Carolina University, Greenville, North Carolina, United States; Undergraduate Student Experiences with Citizen Science Research Coordination Network; North Carolina State University, Raleigh, North Carolina, United States; Undergraduate Student Experiences with Citizen Science Research Coordination Network

**Keywords:** assessments, cognition, education, environmental science, monitoring or mapping

## Abstract

Citizen science involves the public in science to investigate research questions. Although citizen science facilitates learning in informal educational settings, little is known about its use or effects in postsecondary (college or university) settings. Using a literature review and a survey, we describe how and why citizen science is being used in postsecondary courses, as well as the impacts on student learning. We found that citizen science is used predominantly in biologically related fields, at diverse types of institutions, to improve student engagement and expose students to authentic research. Considerable anecdotal evidence supporting improved student learning from these experiences exists, but little empirical evidence exists to warrant any conclusion. Therefore, there is a need to rigorously assess the relationship between citizen science participation and postsecondary student learning. We highlight considerations for instructors planning to incorporate citizen science and for citizen science projects wanting to facilitate postsecondary use.

Citizen science, which we use in this article as an umbrella term to include community science and other participatory science approaches (Cooper et al. 2021), involves nonscientists in the processes of science to advance scientific knowledge or community action (NASEM 2018). Even though citizen science has occurred in various forms for hundreds of years, the formalization of citizen science as a field of study is much more recent (Jordan et al. 2015, NASEM 2018). Public participation in science has grown over the past 25 years because of the scientific need to address large-scale scientific questions that require the efforts of great numbers of individuals, interest in harnessing the public's focus on local scientific concerns in a community, technological advances that make collaborative work and affordable environmental monitoring easier, and the internationalization of citizen science. Although some projects have participants’ learning as a desired outcome, it is sometimes seen as an added benefit instead of a focus of the project (NASEM 2018, Phillips et al. 2018a).

Citizen science originated and continues to be used most prolifically outside of formal educational settings. Research on participant learning through involvement with citizen science has been conducted in informal environments (Brossard et al. 2005, Evans et al. 2005, Jordan et al. 2011, Crall et al. 2012, Price and Lee 2013, Bonney et al. 2016, Merenlender et al. 2016, Ballard et al. 2017, He and Wiggins 2017, Lynch et al. 2018, Halliwell et al. 2021). The evidence suggests that citizen science in informal settings can enhance the sense of place (Evans et al. 2005, Haywood et al. 2016), expose the participants to scientific tools and practices (Bonney et al. 2016, Merenlender et al. 2016), and increase project-specific disciplinary content knowledge (Brossard et al. 2005, Jordan et al. 2011, Bonney et al. 2016, Merenlender et al. 2016, He and Wiggins 2017, NASEM 2018).

Educators at all levels have recognized the potential benefits of citizen science in classroom settings (Tsivitanidou and Ioannou 2020, Abourashed et al. 2021). Although citizen science has been implemented and studied primarily in primary and secondary education (Paige et al. 2015, Shah and Martinez 2016, Schuttler et al. 2018, Tsivitanidou and Ioannou 2020), it had eventually filtered to instructors and their students in postsecondary education (e.g., colleges and universities). The participant benefits documented through citizen science in informal educational settings may be enhanced or reduced in formal education settings (such as courses). Formal educational settings can provide additional resources and structure, but the students may be motivated by grades instead of voluntary intrinsic interest. For example, learning goals involving conceptual change may be achievable in formal educational settings more than in informal settings because of the possibility of more sustained active facilitation and scaffolding of opportunities, but little research has directly tested such hypotheses (NASEM 2018).

Postsecondary education in the United States consists of 2-year colleges that serve as trade schools or that award associate's degrees, 4-year colleges that award bachelor's degrees, and universities that award bachelor's degrees, master's degrees, and possibly doctoral degrees. Carnegie classifications (https://carnegieclassifications.iu.edu) are a common way to describe institutional typology in US higher education on the basis of the number and types of degrees they award and their level of research intensity. Institutions can also be designated as a minority serving institution (MSI; www.doi.gov/pmb/eeo/doi-minority-serving-institutions-program) if they enroll a significant percentage of students from minority groups. Although case studies exist in the literature of citizen science being used in a variety of institutional types, no comprehensive effort seems to have been published (in English) to synthesize where and how—or even how frequently—citizen science is being incorporated into postsecondary courses.

We present a description of the use of citizen science with students in postsecondary education, primarily in the United States, and its potential influence on student learning outcomes. Student learning outcomes were defined broadly as skills, competencies, and knowledge that students have achieved or changes in student feelings or attitudes. We reviewed the published literature and conducted a survey of instructors (i.e., anyone teaching in a postsecondary setting) to address four research questions: What types of institutions (i.e., colleges and universities) and courses are using citizen science with students? What types of citizen science projects are being used in these settings, and in what ways are students participating in them? What learning objectives are instructors hoping to address through their use of citizen science, and what evidence supports student learning from their participation? What challenges did instructors perceive, and what resources may ameliorate the use of citizen science in postsecondary courses? Our findings highlight considerations and opportunities for instructors and project managers to reduce barriers to the use of citizen science in postsecondary courses.

## Literature review

**Figure 1.**
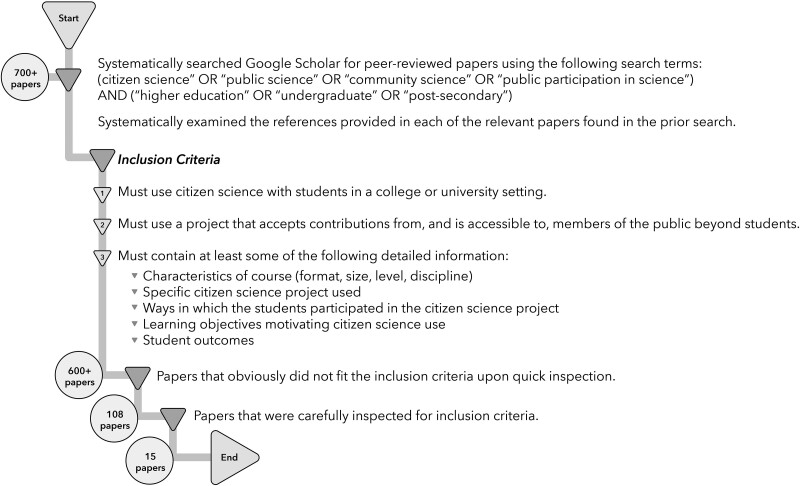
The search methods and inclusion criteria for the literature review.

During summer 2020 and again in summer 2021, we compiled relevant papers we knew from the primary literature and systematically searched Google Scholar for papers using the following search terms in combination: (“citizen science” OR “public science” OR “community science” OR “public participation in science”) AND (“higher education” OR “undergraduate” OR “post-secondary”). Although this search method accessed international publications, it is primarily based on English-language publications from the United States. It is possible that sources such as evaluation reports to grant agencies contain relevant information, but only published peer-reviewed papers were considered for this literature review. This initial search yielded over 700 papers. Over a period of approximately 2 months, we examined each of those papers to determine whether they met our inclusion criteria (see below and figure 1).

Next, we examined the references provided in each of the relevant papers found in the prior literature searches. In total, only 15 papers fit our inclusion criteria as of August 2020 (supplemental table S1). Although the small number of papers remaining after our search seems surprising, it agrees with the findings of prior studies. In a recent paper examining the scientific benefits of using citizen science in schools of any level (primary, secondary, postsecondary, university), Abourashed and colleagues (2021) conducted a literature search and out of more than 4000 results, and they found only 23 studies on life sciences.

For a paper to meet our inclusion criteria, it needed to include the use of citizen science with students in a postsecondary setting, a project that accepts contributions from and is accessible to members of the public beyond students, and at least some details (but not all) about course characteristics, the specific citizen science project used, ways in which students participated in the project, learning objectives motivating the use of citizen science, or the results of assessments of student outcomes (figure 1). For our purposes, we defined citizen science projects as those that accept contributions from and were accessible to members of the public beyond students. Therefore, papers describing course-based undergraduate research experiences (https://serc.carleton.edu/curenet/index.html) that were shared among multiple institutions but where the data and project were not accessible outside of these courses did not fit our inclusion criteria. The use of databases, such as iNaturalist, was considered to fit our definition of citizen science. Review papers that listed citizen science projects that have been used with postsecondary students but that did not include at least some details listed in the third inclusion criterion were excluded.

The focus for most of the papers in the final pool was either the scientific results from data collected by students (e.g., Cosentino et al. 2014, Riley et al. 2020) or encouragement for instructors who may want to add citizen science to their courses (e.g., Voss and Cooper 2010, Oberhauser and LeBuhn 2012, Surasinghe and Courter 2012, Kridelbaugh 2016, Hardy and Hardy 2018). Only a small subset of the 15 papers was focused, even partially, on the results of assessments of student outcomes from their participation in citizen science. Therefore, we were unable to quantitatively evaluate assessment results from the literature.

On the basis of a final pool of only 15 papers that did not all contain the same types of information from our third inclusion criterion, we did not undertake a full meta-analysis. Instead, two individuals coded each of these 15 papers with respect to the institution and course attributes on the basis of information in the paper or in publicly available sources (e.g., university student enrollment numbers on websites), and any areas of disagreement in interpretation were discussed and resolved. The citizen science projects were categorized by discipline using the coding scheme formulated by Allf (Bradley Allf, North Carolina State University, personal communication, 2 December 2020) and Caren Cooper (an author of the present manuscript). Those attributes were then imported into R (R Core Team 2020) to be described. In addition, three individuals read all the papers and agreed on broad qualitative themes that emerged from the final set of papers.

## Survey

We developed a survey to capture information about how citizen science was being used in postsecondary courses that may not have been accessible through our literature search methods. The purpose of the survey was to collect information on the institutions and courses using citizen science, the citizen science projects or databases being selected, and the motivations and outcomes of the student citizen science participation. The survey questions complemented the data extracted from the literature review in which the respondents were able to report the use of up to three citizen science projects in up to three different courses. Most of the questions involved selecting among discrete answer choices, but there were a small number of open-ended questions (supplemental file S2). The survey was reviewed by six professionals for content and clarity, and then revisions were made. It was approved as exempt by the East Carolina University Institutional Review Board (project no. UMCIRB 21–001,211). A link to the Qualtrics survey was distributed to more than 10,000 email addresses on multiple listservs (see below) and through professional contacts in January 2020. In spring 2020, we added two COVID-specific questions to the survey to capture additional data after the widespread transition to remote learning and then redistributed the survey using the same distribution outlets. The individuals on these listservs were asked to complete the survey if they were willing to provide at least some details about a course they taught recently that fit the same inclusion criteria as the literature review. Overall, 102 instructors responded to the survey with information beyond contact information. On closer inspection, 23 of the instructors either did not provide usable data or reported activities that did not fit our inclusion criteria. Therefore, 79 instructors from 62 unique institutions provided usable data. We will refer to these instructors as our survey respondents.

The instructor survey was sent to listservs from the Ecological Society of America (i.e., ECOLOG-L), the Citizen Science Association, and the Society for the Advancement of Biology Education Research. ECOLOG-L subscribers include ecologists and people interested in environmental fields. This listserv was selected because the majority of citizen science projects are related to environmental fields (Abourashed et al. 2021). The Citizen Science Association listserv subscribers include postsecondary instructors but also citizen science project staff, primary and secondary instructors, and other individuals with an interest in citizen science. Finally, the Society for the Advancement of Biology Education Research listserv was selected as individuals with experience in testing innovative teaching approaches subscribe, along with postsecondary instructors. Each of these listservs has subscribers from outside the United States but most heavily represent United States–based participants. We recognize that these distribution outlets are biased toward biological fields, and our results are mainly representative of the use of citizen science in biological, ecological, or environmental postsecondary courses. Because a large proportion of citizen science work is concentrated in these fields, our study makes a meaningful contribution to understanding how citizen science is being used in postsecondary settings. These findings certainly underestimate the use of citizen science in postsecondary settings but are the most comprehensive effort to date that describes the use of citizen science in these settings.

We coded both pre- and post-COVID data sets and contacted some respondents, as necessary, to clarify their responses. We also accessed publicly available data to code additional information about United States–based institutions, such as Carnegie classifications and lists of MSIs. The coded attributes were imported into R (R Core Team 2020) for description. Open-ended survey questions about the benefits instructors perceived from the use of citizen science, along with barriers or challenges, and resources that would have been beneficial to instructors implementing citizen science were coded and analyzed qualitatively for themes.

## Literature review and instructor survey results

The results from the literature review and the instructor survey were very similar. Therefore, we present the results of these two data sources together, except in cases in which the data to address a question were available only from the instructor survey.

### Institutions and courses using citizen science

**Figure 2.**
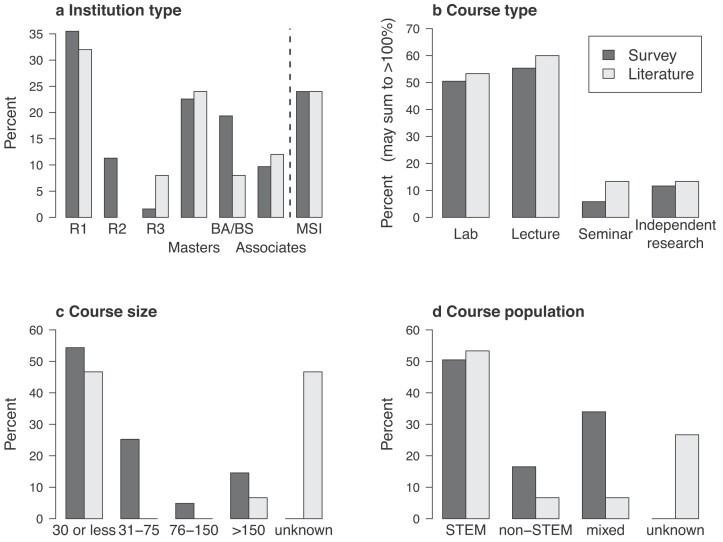
Characteristics of the institutions and courses using citizen science, as they were reported in the survey of instructors (the black bars) or literature review (the grey bars). The y-axis value refers to the percentage of overall institutions (or courses) of that particular type. (a) Carnegie designations of reporting institutions, in which R1, R2, and R3 are doctoral universities of varying degrees of research intensity, master's colleges and universities award at least 50 master's degrees and fewer than 20 doctoral degrees in a given year, baccalaureate colleges award primarily baccalaureate degrees, and an associate's college does not award degrees higher than an associate's degree. Minority serving institutions (MSIs) are categorized with the prior classifications in addition to the MSI status. (b) Course types, where courses may be designated in more than one category (e.g., lecture and lab combination course). (c) Course sizes, which were not always reported in the published papers (i.e., unknown). (d) Course populations, which, again, were not always reported in the published papers (i.e., unknown).

A variety of institution types used citizen science in postsecondary courses. The literature review included courses mainly from the United States, but one was from Austria (Heigl and Zaller 2014), and one was from Australia (Mitchell et al. 2017). In addition, one paper had data from both the United States and Ireland (Phillips et al. 2018b). Most of the respondents from the instructor survey were from the United States, but at least one instructor each responded from Canada, England, and New Zealand. The highest percentage of United States–based papers and survey responses came from institutions that had a Carnegie classification of doctoral highly research intensive (R1), but master's, baccalaureate, and associate's institutions were also represented (figure 2). The institutions included those with enrollments greater than 20,000 students (44% and 42% of institutions from literature review and survey, respectively) and those with enrollments less than 10,000 students (36% and 47% of institutions from literature review and survey, respectively). Institutions with enrollments less than 5,000 students were represented much more frequently in the instructor survey than in the published literature. Approximately a quarter of these institutions (24% from both the literature review and instructor survey) were designated as MSIs, and most of the institutions were public (92% and 76% from the literature review and survey, respectively). On the instructor survey, most of the institutions (94%) had only a single instructor respond as a user of citizen science in their courses, and most of the institutions (71%) had only one course reported to include citizen science. North Carolina State University (the affiliation of two of the present authors), which has established a citizen science campus, was an outlier, with 11 unique instructors responding about the use of citizen science in 13 different courses.

Diverse course types used citizen science with postsecondary students. Citizen science was reported to be used most in lecture courses, lab courses, and combined lecture–lab courses. The remaining courses included seminars or independent research (figure 2). Although larger courses (with more than 150 students per course section) were represented, over half of the courses using citizen science projects enrolled fewer than 30 students per course section (figure 2). Citizen science projects were used more commonly in introductory courses than in advanced courses (80% and 60% of courses from literature review and survey, respectively).

In the United States, students in bachelor's degree programs select a field of study, which is called their major. The target student audience in courses incorporating citizen science was generally those studying science, technology, engineering, or mathematics (i.e., STEM majors) or a mixture of STEM and non-STEM majors, as opposed to students pursuing non-STEM majors (figure 2).

On the instructor survey, most of the instructors (79%) reported using citizen science projects in a single course, with the remainder of the instructors incorporating citizen science projects into multiple courses. As the course instructors shifted to alternate delivery methods in response to the COVID pandemic, they continued to use citizen science projects in their classes. Among the 50 courses for which the delivery method was reported on the instructor survey, almost half (46%) were fully or partially online.

### Projects being used and ways students are participating

**Figure 3.**
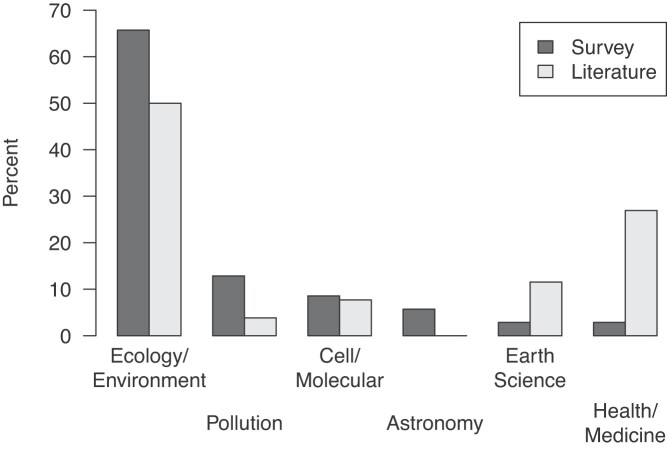
The topics of the citizen science projects reported in the survey (the black bars) and literature (the gray bars).

Most of the courses involved students in only one citizen science project, and many different projects were used across courses. For example, 74 unique projects were reported on the survey, with 77% of those projects being used in only one course (supplemental table S3). On the instructor survey, the only projects reported to be used in more than six courses were the Global Learning and Observations to Benefit the Environment Program (GLOBE) and the iNaturalist platform. Most of the project topics were classified as ecology and environment (62% on survey, 50% in the literature), with a variety of other fields represented in relatively low numbers of courses (figure 3). Projects categorized as health and medicine were more prevalent in the literature than the survey, possibly because of bias in the listservs through which we distributed the instructor survey (figure 3). Finally, 26% of the projects reported in the survey take place entirely online.

**Figure 4.**
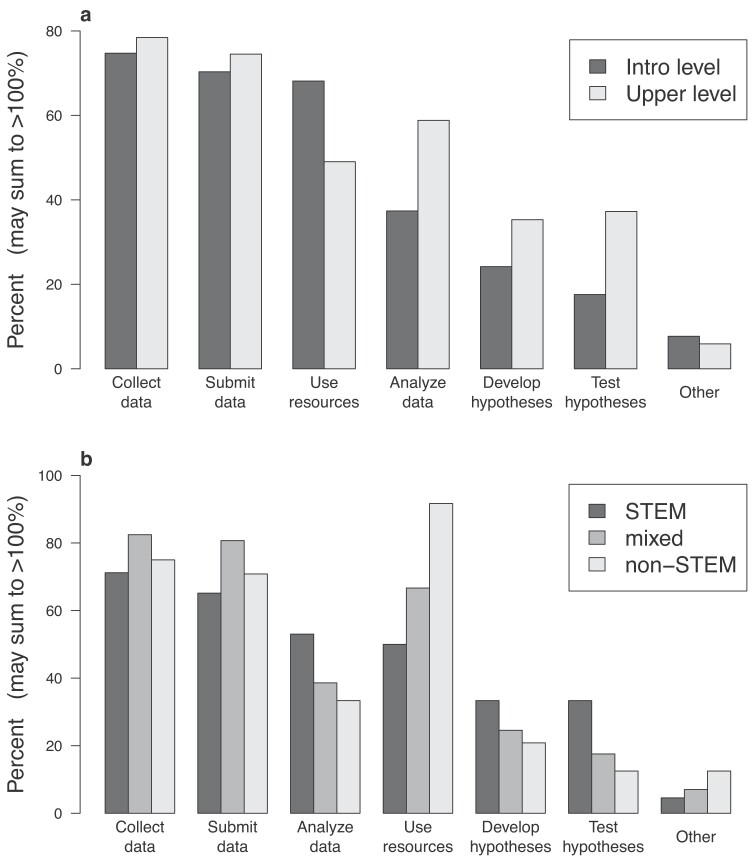
Ways students participated in citizen science in their postsecondary course, by course level (a) and whether the course was for STEM majors, non-STEM majors, or a mix of both (b).

Detailed information on how the students participated in the citizen science project was not available in most of the published papers. The survey respondents, however, were asked to identify how they implemented citizen science with their students. Most of them reported their students participating in multiple aspects of a citizen science project (figure 4). The most common way students participated in citizen science, at all levels, was by collecting and submitting data to the project. Participation was similar in the introductory level and upper-level courses, with the exception that the introductory courses were focused slightly more on having the students use training resources provided by the project (e.g., online quizzes to practice taxonomic identifications), whereas the upper-level courses had a greater emphasis on testing hypotheses and analyzing data (figure 4a). Similarly, nonmajor courses used project training resources more than courses for STEM majors did, whereas courses for STEM majors more frequently involved the students developing and testing hypotheses and analyzing data (figure 4b).

### Learning objectives being addressed and evidence to support student learning

**Figure 5.**
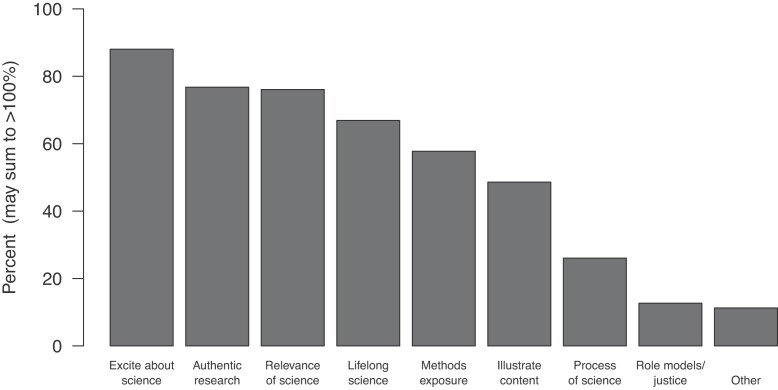
Learning objectives that were identified by survey respondents as motivating their use of citizen science. Multiple learning objectives were generally reported for each course. The full x-axis labels, in order, are “to get students excited about science,” “to expose students to authentic scientific research,” “to show students the relevance of science to the real world,” “to introduce students to a means of contributing to science and society after graduation,” “to reinforce a specific aspect of the scientific process, e.g., sampling, graphing,” “to illustrate specific content covered by the project,” “to allow students to go through the entire scientific process with an independent question,” and “to show diverse role models or discuss issues of social justice.”

The published papers generally did not specify the instructors’ learning objectives for using citizen science. In the survey, however, the respondents were asked to select any relevant learning objectives motivating their use of citizen science from a list of options or to write in additional items. Multiple learning objectives were generally reported for each course. The most frequently selected options were to get students excited about science, to expose students to authentic scientific research, and to show students the relevance of science to the real world (figure 5). The free-text additions to this question varied, but there were multiple responses that could be classified as modeling the use of citizen science for K–12 (primary and secondary) teachers in training.

The published literature provided little data on the student learning gains from their participation in citizen science. Only two of the papers (Vitone et al. 2016, Mitchell et al. 2017) assessed student learning beyond student perceptions or reflections. Of these, Vitone and colleagues (2016) saw no change in content knowledge and mentioned the need for more sensitive assessment instruments, whereas Mitchell and colleagues (2017) did not describe the results of their assessment for student learning. The authors of most of the papers, however, reported many substantial benefits to their students, on the basis of anecdotal evidence and the perceptions of the students and instructors. The perceived benefits included positive student feelings about participating in citizen science, increased student engagement, and improved awareness of societal issues (e.g., native biodiversity, road kills in the environment).

**Figure 6.**
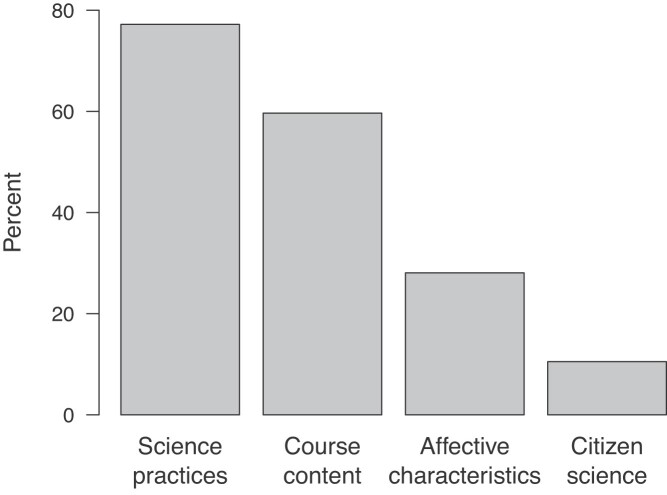
The focus of the assessments instructors reported (on the survey) giving their students related to their citizen science participation. “Affective characteristics” include aspects such as student motivation or science identity. “Citizen science” includes student familiarity with, or interest in, citizen science. Instructors could select more than one topic.

Out of the 57 courses reporting assessment information on the instructor survey, most assessed one or more specific scientific practices or course content. Although making science more engaging and relevant to the real world were two of the most cited learning objectives for using citizen science in a class, few of the instructors measured affective characteristics such as motivation or science identity. A small percentage of the instructors assessed their students’ familiarity with or interest in citizen science (figure 6). These course assessments were overwhelmingly instructor generated. Very few instructors used any published or publicly available instruments (16%) or instruments provided by the citizen science project (5%). The assessments were frequently in the form of an assignment (43%) or formal paper, poster, or oral presentation (40%), although tests and quizzes, surveys, and student reflections were also used regularly (21%, 21%, and 19% of courses, respectively). Less frequently, the instructors used metrics of the amount of time spent or the quantity of the data collected or metrics associated with data quality as judged by an expert (16% and 2% of courses, respectively) to assess their students.

Similar to those in the published literature, the respondents to the instructor survey generally did not report any results from formal assessments of students, but the open-ended responses from 86% of the respondents described their perceived benefits from incorporating citizen science projects into their courses. These open-ended responses broadly fell into four themes (table 1): affective benefits (related to feelings or attitudes), improved scientific practices or content knowledge, implementation benefits (e.g., helpful training materials, topics at the correct level for the students, project was easy to implement), and exposure to citizen science. For example, over half of the instructors (53%) reported on the survey that their students’ engagement was improved by the use of citizen science in class.

**Table 1.** Primary benefits and challenges identified by survey respondents related to incorporation of citizen science projects into postsecondary courses.

Theme	Specific perceived benefits	Percentage of instructors	Number of instructors
Affective	Increased student engagement, enjoyment, or interest in science	53	36
Scientific practices or content	Students contributed to authentic scientific research or interacted with project scientists	49	33
	Students gained experience with using databases and technology	24	16
	Students learned a specific scientific practice or content	16	11
Implementation	The project was easy to implement or fit well with the course subject, format, class size or experience level of the students	15	10
	Students were able to participate in hands-on, experiential learning, often in an outdoor setting	15	10
Exposure to citizen science	Students learned about the benefits of citizen science	9	6
	Specific perceived challenges		
Logistical	Project required too much time or did not fit well with the course schedule or academic calendar	33	21
	Difficulty with finding or accessing relevant projects, publications, instructional materials, or assessment tools	20	13
	Difficulty interacting with project managers, database, website, or community partners	17	11
	Difficulty with implementing project because of technology or internet issues, travel requirements, or inability to access to off-campus sites	13	8
	A lack of funding or administrative or departmental or staffing support	9	6
Affective	A lack of student interest or investment in the project; the students did not see relevance of project or how their contributions would be used	31	20
Data Issues	Concerns about data quality, including the students’ lack of confidence in identification skills	14	9
	Difficulty with data analysis and interpretation	11	7

*Note:* These benefits and challenges were identified by instructors through their own perceptions and not from formal assessment results. Instructors could report more than one benefit or challenge. A total of 68 instructors provided open-ended responses about benefits, whereas 64 provided open-ended responses about challenges.

### Challenges and resources needed

Few of the published papers discussed the challenges experienced when incorporating citizen science projects into their courses, but 81% of the survey respondents provided open-ended descriptions of their challenges. These open-ended responses broadly fell into three themes (table 1): logistical challenges, affective challenges, and problems related to data quality, analysis, and interpretation. Most of the respondents (72%) reported at least one challenge related to the logistics of implementing citizen science projects into their courses. The most common challenge identified within this category (by 33% of instructors) was that the project required too much time or did not fit well with the timing of the course schedule or academic calendar. Other common challenges (identified by 20% of instructors) were difficulties with finding and accessing relevant projects, publications, or instructional materials. Nearly a third of the instructors (31%) reported affective challenges (relating to feelings and attitudes), in which the students were not interested in the project or did not see its relevance. Finally, some instructors reported that they or their students lacked confidence in the quality of the data collected, especially when the students felt uncomfortable with their ability to use field guides to correctly identify organisms.

A lack of sufficient resources to assist in finding and implementing citizen science projects in a class was listed as a challenge by 20% of the respondents on the instructor survey. When asked what resources would be beneficial, several were highly requested (table 2), such as implementation examples and lesson plans. In addition, 11% of the respondents selected “other” and wrote in an open-ended text response. These open-ended responses could be categorized into three groups: funding sources to implement citizen science in a course, additional learning tools to help students learn necessary skills (e.g., taxonomic identification), and lists of citizen science projects that meet global goals (e.g., United Nations sustainable development goals).

**Table 2.** Resources desired by survey respondents to facilitate the incorporation of citizen science projects into postsecondary courses.

Resources wanted (ranked in order)	Percentage of instructors	Number of instructors
Examples of how citizen science is being used in higher education	88	62
One comprehensive website compiling existing resources for the use of citizen science in higher education	77	54
University-level lesson plans for specific citizen science projects	69	48
Course learning objectives mapped to specific citizen science project characteristics	61	43
Guidance finding validated evaluation instruments	56	39

*Note:* Instructors could select more than one resource.

## Implications of these findings

These results clarify how citizen science is being used in post-secondary settings and inform the discussion of best practices for its use.

### What types of institutions and courses are using citizen ­science?

Data from both the literature review and the survey reveal that citizen science use in higher education occurs across nearly all institution types, course sizes, and instructional formats. The many kinds of citizen science projects, coupled with the diverse ways they can be incorporated into classes, provides considerable flexibility for instructors when incorporating citizen science. The ability of citizen science to be implemented with students in an online environment makes it a particularly desirable option for instructors teaching during a disruption such as the COVID-19 global pandemic. For example, some of the instructors reported replacing in-person laboratory exercises with student data collection for online citizen science projects or used citizen science data sources to have their students practice analyzing and interpreting data in place of collecting data. Although featuring in-depth case studies of how citizen science has been used in postsecondary courses is beyond the scope of this article, examples can be found in the published literature (e.g., those listed in supplemental table S1).

### What projects are being used and in what ways are students participating?

Many different citizen science projects were being used in postsecondary, mostly through student participants collecting data and submitting it to the project. Additional research is needed to define the project characteristics that make a project attractive for instructor use, but the high level of use of training materials supplied by a project suggests that the instructors’ choice of projects to use with their class may be influenced by the training resources a project makes available. Citizen science projects may be able to increase rates of adoption by reaching out to postsecondary instructors or making training resources and educator resources (e.g., lesson plans) available on their websites or a third-party platform such as SciStarter (https://scistarter.org) or Zooniverse (www.zooniverse.org). For example, helpful project resources could include apps designed to test participants on necessary taxonomic identification, videos of data collection methods, instructor materials, or well-designed platforms for visualizing data from multiple locations and time periods. These and other emerging technologies bring more tools of science inquiry to the public as well as diverse postsecondary science courses.

### What learning objectives are instructors trying to address through their use of citizen science and what evidence supports student learning from their participation?

Instructors were motivated to include citizen science in a course primarily as a means of increasing student engagement, exposing students to authentic research, and contributing to research of global or local relevance. There were no apparent differences in how students participated in citizen science projects on the basis of the instructor's reported learning objectives. This uncoupling of student tasks and learning objectives may be problematic as participant outcomes have been found to differ on the basis of the tasks they complete (Lin Hunter et al. 2020).

Most assessments of student learning outcomes being used in the literature and reported on the instructor survey are qualitative reflections by students, results from unvalidated surveys of self-reported student interest or engagement, or instructor-generated assignments or papers on scientific practices or course content. Virtually none of the results of these assessments have been reported (but see Vitone et al. 2016 and Mitchell et al. 2017). The paucity of papers or survey responses providing assessment data related to educational outcomes of postsecondary students’ participation in citizen science in a formal setting makes any definitive conclusions about impacts on learning outcomes premature and provides a clear avenue for future research.

The authors of relevant papers and the instructor survey respondents, however, reported many substantial benefits to their students, on the basis of anecdotal evidence and their perceptions. These perceived benefits largely match the learning objectives instructors had for using citizen science in their courses, such as getting students excited about science. Rigorous assessment of citizen science in postsecondary courses is still needed to support or refute these perceptions. As most postsecondary instructors are not interested or skilled in conducting educational research or program evaluations, the outcomes of most courses that use citizen science will continue to be untested. Valuable data could be gathered, however, by instructors partnering with educational researchers or citizen science evaluation specialists with the necessary expertise.

### What challenges did instructors perceive?

The challenges that instructors reported when using citizen science in their courses were related to logistics and student affect. Projects that are easily searchable and include instructor resources on platforms such as SciStarter (https://scistarter.org) and Zooniverse (www.zooniverse.org) can reduce the logistical challenge of finding and accessing relevant projects, publications, or instructional materials. In addition, projects that can be divided into smaller modules or conducted in different seasons may help reduce some of the stated logistical challenges some instructors reported.

The challenges regarding student affect were mainly reported as a lack of interest in citizen science by some students. Although the instructors generally perceived the use of citizen science to help student interest, it was not uniformly appealing to all of their students. Student interest may vary by project, how the project is used, and student population. To increase the likelihood that postsecondary students will be engaged in citizen science and see it as a valuable part of the course, instructors need to scaffold their use of the project within the course curriculum to demonstrate its fit with course objectives and articulate the project's scientific or social relevance (Abourashed et al. 2021).

### What ethical considerations exist?

Another challenge reported to using citizen science in postsecondary courses was that instructors and students sometimes lacked confidence in the quality of data being collected. Two specific data quality concerns are most salient to the use of citizen science with students: research misconduct and a lack of expertise.

Part of exposing students to authentic scientific research should include understanding basic research ethics including the three components of research integrity: no data falsification, data fabrication, or plagiarism (Anderson et al. 2013). Student codes of conduct can be extended to include research integrity when citizen science is assigned.

In addition, some citizen science projects may not have the desire or resources available to accommodate volunteers with little expertise, whereas others require no prior experience or provide no training for volunteers. When instructors are selecting projects to meet learning objectives, they should consider whether students have, or can easily gain, the necessary skills or expertise to contribute valuable, reliable data. Making relevant training resources available and incorporating data quality checks may be critical to improving the student experience. When protocols are explicit, data quality from students has been found to be generally moderate to high or high quality (von Konrat et al. 2018, Hurlbert et al. 2019, Abourashed et al. 2021).

A related challenge that was not reflected in the instructor survey responses but that is relevant to any discussion of the use of citizen science in postsecondary courses is the ethical implications of requiring students to participate (Bowser et al. 2017). Requiring students to use certain apps or websites can reveal information about the students’ locations and may require them to divulge private information to complete their assignment and receive a grade. This discussion of ethics is ongoing in the citizen science community, but instructors need to consider the implications carefully. As an alternative, some citizen science projects allow instructors to create dummy accounts for students to use that are not linked to information about specific students, whereas others allow users to obscure their locations. Therefore, instructors may need to provide instructions on how to change privacy settings within these project technologies (see the supplement in Hitchcock et al. 2021 for more information on ethical and accessibility issues).

### What resources may ameliorate the use of citizen science in postsecondary courses?

When asked what resources would be beneficial in ameliorating these challenges, examples of how citizen science could be implemented in a course and lesson plans were highly requested. Because examples of the use of citizen science in higher education are not commonplace in the published literature (but see supplemental table S1), it is not surprising that faculty would like to see more examples of the use of citizen science in a formal postsecondary setting. This need could be met by more instructors publishing case studies of their experiences with citizen science in the peer reviewed literature. Sites such as SciStarter and CourseSource (www.coursesource.org) can provide instructors with strategies of student engagement (e. g. Lin Hunter et al. 2020), as well as access to lesson plans and other resources for specific citizen science projects. Finally, networks such as the National Science Foundation funded Undergraduate Student Experiences with Citizen Science (USE Cit Sci, use-cit-sci-network.org) research coordination network (of which the present authors are members) are working toward more systemic change through increased collaboration between various citizen science stakeholders. The Network will also compile links to existing resources and create new resources, such as ideas for evidence-based strategies for assessing citizen science participation in formal higher education settings and will disseminate them widely.

### How can the field of citizen science benefit from postsecondary course participation?

Although this article's focus is on impacts on postsecondary student participants and instructors, these results indicate that the field of citizen science also could benefit from having postsecondary students participate. Incorporating citizen science into postsecondary courses is a particularly effective way of introducing citizen science to students and broadening participation in citizen science. Instructors from both the published literature and survey reported that most students had little or no familiarity with citizen science before their exposure to it in class. As citizen science becomes increasingly well known in society, this situation may change. For now, though, incorporating citizen science into postsecondary courses may serve as an important way to introduce citizen science to new populations. Approximately a quarter of the institutions represented in both the literature review and survey data are MSIs. Because citizen science participants have been found previously to be overwhelmingly white, college educated, and middle income (Dickinson and Bonney 2012, Cooper et al. 2021), the addition of participants from MSIs (who are likely to have sizable percentages of students of color) can introduce valuable diversity to the pool of participants. Some of these students will likely continue in citizen science after their course ends (Hitchcock et al. 2021), making courses a potentially effective way to broaden participation in citizen science long term.

### How do the results from postsecondary courses compare to prior literature from informal learning environments?

Although some similarities are apparent in the use of citizen science in informal and formal educational settings, important distinctions exist. Informal and formal educators often use citizen science to meet similar objectives: participant engagement, real world science, and societal contributions. Also, both settings struggle with the need to describe and formally evaluate the participants’ learning outcomes (Bonney et al. 2016, Phillips et al. 2018a). Despite these similarities, an important difference remains: participant choice. In formal courses, students likely have little or no autonomy to engage in a citizen science project. Therefore, a subset of students will likely have quite different motivations and outcomes compared to enthusiastic volunteers that choose to do a citizen science project in an informal setting. This distinction was reflected in our survey results (table 1), in which one of the barriers identified by instructors was a lack of interest by some students. Instructors may be able to increase student interest by making it obvious how involvement in the project contributes to course learning objectives and answers important research questions. In addition, allowing students to choose among a small number of citizen science projects with differing topics or choosing projects with obvious community relevance may lead to greater student interest. According to self-determination theory (Deci and Ryan 2008), students will learn more if they progress in self-determination from extrinsic to intrinsic motivation. Three conditions have been identified that are associated with gains in self-determination: autonomy support, involvement, and structure (Guay et al. 2008). Research is needed to determine how to design higher education experiences with citizen science to meet these needs. Even with these efforts, however, the extrinsic motivation of course grades will be an important difference from the intrinsic motivation of most informal science participants. Therefore, the use of citizen science in higher education versus informal settings provides an opportunity to examine the links between motivations and learning.

### Need for postsecondary instructors and citizen science staff to collaborate.

The literature search and survey revealed little evidence of interactions between citizen science project staff and instructors and students who may be participating, despite several instances in which a desire for more interaction was stated. Many managers of the citizen science projects being used in higher education likely do not know their projects are being used in this setting as instructors often find projects on their own and do not communicate with project staff. This lack of interaction represents a lost opportunity for both groups. Projects could benefit as instructors reported that they or their students sometimes had suggestions to make project interfaces more user friendly. In addition, projects may want to know whether faculty are validating the accuracy of data being entered by students into a project database and removing duplicate entries, resulting in improved data quality. In return, instructors could benefit from the expertise of the project staff to facilitate implementation of citizen science activities in their courses and from the inclusion of interactions between project scientists and students to enhance the learning experience.

Although instructors reported a desire for more interaction with citizen science project staff, some examples of such interactions exist. The projects that began locally to the postsecondary institution or are very community based were more likely to interact with instructors at those locations. For example, one instructor mentioned that project staff visited their class, and the students appreciated the interaction. Instructors wishing to have more interaction with citizen science project staff may want to consider choosing a local project. These opportunities can be expanded to full cooperation between students and a community-based project (Wilderman 2007, Bonney et al. 2009), as a way of involving students in community-engaged learning experiences. These experiences emphasize the broader context of scientific pursuits as well as the collaborative and interdisciplinary state of science (Malotky et al. 2020), addressing multiple competencies from the Vision and Change report that provides a call to action for postsecondary biology education (AAAS 2011).

## Conclusions

The versatility of citizen science for use in diverse class formats, disciplines, and institution types makes its use appealing, but there is much we still do not know about its impact on student learning outcomes in higher education. Increasing dialogue among instructors using citizen science, education researchers, students, and citizen science project staff will allow us to better understand the strengths and limitations of incorporating citizen science in postsecondary courses and provide a toolbox of resources for successful implementation. In addition, increased dialogue among various stakeholders may improve the generation of scientific data. Efforts such as the National Science Foundation funded USE Cit Sci Network will be instrumental in facilitating the engagement of students in authentic science practices in both face-to-face and online settings, the improvement of student learning outcomes, and the broadening of participation in science.

## Supplementary Material

biab125_Supplemental_File

## Author Biographical

Heather D. Vance-Chalcraft (vancechalcrafth@ecu.edu) is an assistant professor of biology and Michelle Anne Reyes is a graduate student at East Carolina University, in Greenville, North Carolina, in the United States. Allen H. Hurlbert is a professor of biology at the University of North Carolina at Chapel Hill, in Chapel Hill, North Carolina, in the United States. Jennifer Nesbitt Styrsky is an assistant professor in the Westover Honors College and in the School of Sciences at the University of Lynchburg, in Lynchburg, Virginia, in the United States. Terry A. Gates is an assistant teaching professor in the Department of Biological Sciences and Caren B. Cooper is an associate professor in the Leadership in Public Science Program and leads the Citizen Science Campus program at North Carolina State University, in Raleigh, North Carolina, in the United States. Gillian Bowser is an associate professor in the Department of Ecosystem Science and Sustainability at Colorado State University, in Fort Collins, Colorado, in the United States. Colleen B. Hitchcock is an associate professor in the Biology Department and in the Environmental Studies Program at Brandeis University, in Waltham, Massachusetts, in the United States. All of the authors are affiliated with the Undergraduate Student Experiences with Citizen Science Research Coordination Network.
