# Supplementary material for: Citizen Science in Postsecondary Education: Current Practices and Knowledge Gaps
Source: Bioscience. 2022 Jan 5;72(3):276–88. doi: 10.1093/biosci/biab125 (PMC8888125; doi:10.1093/biosci/biab125)
Supplement: biab125_Supplemental_File [file biab125_supplemental_file.docx]

**SUPPLEMENTAL TABLES – VANCE-CHALCRAFT ET. AL.**

**Supplemental Table 1**. List of papers included in the literature review.

| **Papers included** | |
| --- | --- |
| Andrews S, Ho NKP. 2018. Barcoding a lionfish’s last meal: A citizen science research project for the classroom. National Marine Educators Association:7. |  |
| Bowser A, Hansen D, He Y, Boston C, Reid M, Gunnell L, Preece J. 2013. Using gamification to inspire new citizen science volunteers. Pages 18-25. Proceedings of the first international conference on gameful design, research, and applications. | |
| Cosentino BJ, et al. 2014. Citizen science reveals widespread negative effects of roads on amphibian distributions. Biological Conservation 180: 31-38. | |
| Guertin L. 2014. Introducing University Students to Authentic, Hands-On Undergraduate Geoscience Research in Entry-Level Coursework. Pages 215-221 in Tong VCH, ed. Geoscience Research and Education: Teaching at Universities. Dordrecht: Springer Netherlands. | |
| Hardy CR, Hardy NW. 2018. Adapting traditional field activities in natural history education to an emerging paradigm in biodiversity informatics. The American Biology Teacher 80: 501-519. | |
| Heigl F, Zaller JG. 2014. Using a citizen science approach in higher education: a case study reporting roadkills in Austria. Human Computation 1. | |
| Kridelbaugh DM. 2016. The use of online citizen-science projects to provide experiential learning opportunities for nonmajor science students. Journal of Microbiology & Biology Education 17: 105-106. | |
| Mitchell N, Triska M, Liberatore A, Ashcroft L, Weatherill R, Longnecker N. 2017. Benefits and challenges of incorporating citizen science into university education. PLoS ONE 12: e0186285. | |
| Oberhauser K, LeBuhn G. 2012. Insects and plants: engaging undergraduates in authentic research through citizen science. Frontiers in Ecology and the Environment 10: 318-320. | |
| Phillips C, Walshe D, O'Regan K, Strong K, Hennon C, Knapp K, Murphy C, Thorne P. 2018. Assessing citizen science participation skill for altruism or university course credit: a case study analysis using cyclone center. Citizen Science: Theory and Practice 3. | |
| Riley NG, Goller CC, Leggett ZH, Lewis DM, Ciccone K, Dunn RR. 2020. Catalyzing rapid discovery of gold-precipitating bacterial lineages with university students. PeerJ 8: e8925. | |
| Surasinghe T, Courter J. 2012. Using eBird to integrate citizen science into an undergraduate ecology field laboratory. Bioscene: Journal of College Biology Teaching 38: 16-20. | |
| Vitone T, Stofer K, Steininger MS, Hulcr J, Dunn R, Lucky A. 2016. School of ants goes to college: integrating citizen science into the general education classroom increases engagement with science. Journal of Science Communication 15: A03. | |
| von Konrat M, et al. 2018. Using citizen science to bridge taxonomic discovery with education and outreach. Applications in Plant Sciences 6: e1023. | |
| Voss MA, Cooper CB. 2010. Using a free online citizen-science project to teach observation & quantification of animal behavior. The American Biology Teacher 72: 437-443. | |

Supplemental File 2. Survey for instructors about citizen science in post-secondary classes.

Survey Flow


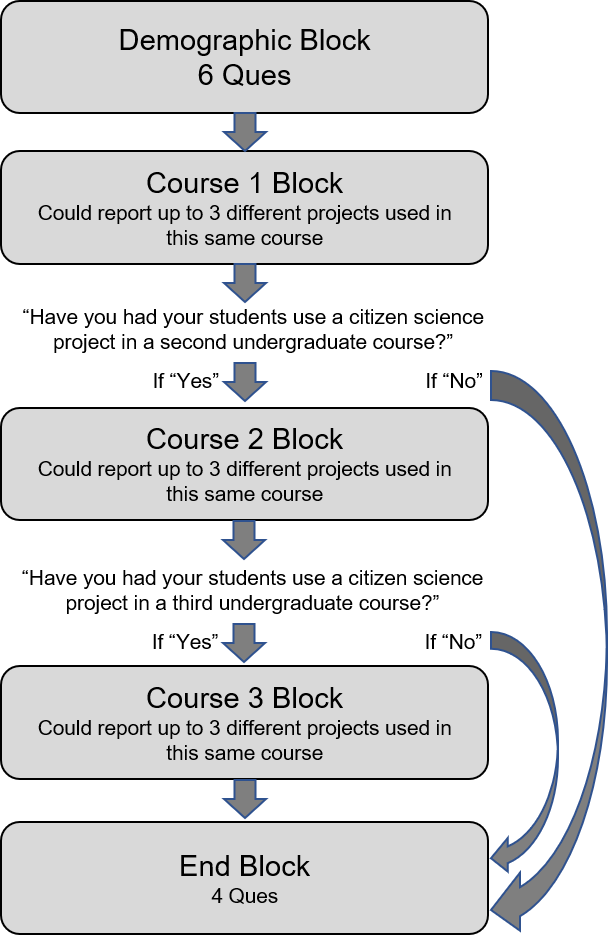


Start of Block: Demographic Block

Q1.1 You are being invited to participate in a **research** study titled “RCN-UBE: The Undergraduate Student Experiences with Citizen Science (USE Cit Sci) Network to transform learning and broaden participation in science” being conducted by Dr. Heather Vance-Chalcraft, a faculty member at East Carolina University in the Biology department.  The goal is to survey up to 500 instructors using citizen science in their courses, interview members of the Research Coordination Network, and review products like curricular materials created during the project. The survey will take approximately 20 minutes to complete, and the focus group interviews will take approximately 45 minutes. It is hoped that this information will assist us to better understand the use of citizen science in undergraduate courses.  Your responses will be kept confidential, and no data will be released or used with your identification attached.  Your participation in the research is **voluntary**. You may choose not to answer any or all questions, and you may stop at any time.  There is **no penalty for not taking part** in this research study.  Please call Dr. Heather Vance-Chalcraft at 252-328-9841 for any research related questions or the Office of Research Integrity & Compliance (ORIC) at 252-744-2914 for questions about your rights as a research participant.

Q1.2
INSTRUCTIONS: The Undergraduate Student Experiences with Citizen Science Network (USE Cit Sci) is collecting information on the use of citizen science in undergraduate courses. We define citizen science broadly as any project that involves non-scientists in the processes, methods, and standards of research, with the intended goal of advancing scientific knowledge or community action (National Academy of Sciences 2018), and with the additional condition that data from the project must be available to individuals outside your class. 
 
Please complete this survey if you have used citizen science (as defined above) in any way in an undergraduate course.  You do not need to have included every step of a citizen science project in your class -- even the use of one small part of a project (e.g., looking at data generated from citizen science) is relevant for this survey. You will be given the opportunity to provide answers on ***up to three citizen science projects in up to three separate courses. Please answer for one specific course at a time***. We appreciate your time to complete this survey!  Feel free to contact Dr. Heather Vance-Chalcraft (vancechalcrafth@ecu.edu) with questions or comments.

Q1.3 First Name

________________________________________________________________

Q1.4 Last Name

________________________________________________________________

Q1.5 Email

________________________________________________________________

Q1.6 Institution

________________________________________________________________

End of Block: Demographic Block

Start of Block: Course 1

Q2.1 Please complete the following questions about a specific undergraduate course in which your students have used part, or all, of a citizen science project that is accessible to individuals outside of your course. You will have an opportunity to report on additional courses later if your students have participated in citizen science in more than one undergraduate course.

Q2.2 Course 1: In what kind of course have you used citizen science? (Check any that apply.)

- Lab course (1)
- Lecture course (2)
- Seminar course (3)
- Independent research course (4)

Q2.3 Course 1: What size was the course in which you used citizen science?

- Class of 30 or less (1)
- Class of 31 to 75 (2)
- Class of 76-150 (3)
- Class size greater than 150 (4)

Q2.4 Course 1: What level of course was it?

- Introductory-level course (1)
- Upper-level course (2)

Q2.5 Course 1: What was the format of the course?

- Face-to-face entirely (1)
- Online entirely (2)
- Hybrid (mix of face-to-face and online) by design (3)
- Started off face-to-face but transitioned to online due to COVID-19 (4)

Q2.6 Course 1: Was the course designed for STEM majors?

- Course for STEM majors (1)
- Course for mixed STEM/non-STEM Majors (2)
- Course for non-STEM majors (3)
- Other: (4) ________________________________________________

Q2.7 Course 1: What is the name of one citizen science project you have you used in this specific undergraduate class? (Note: Your students did not have to complete the project from beginning to end to be included.  Citizen science projects in which only part of the project was included in the course should also be reported.)

________________________________________________________________

Q2.8 Course 1: Website of the citizen science project

________________________________________________________________

Q2.9 Course 1: Did you initially plan to use this citizen science project in your course or did you add it due to the transition to remote learning due to COVID-19?

- Planned to use it in my course all along (1)
- Added it due to COVID-19 transitions (2)

Q2.10
Course 1: In what way did students in your course participate in this citizen science project? (Check any that apply)

- Students used training resources provided by the citizen science project (1)
- Students collected data using the project's protocols (2)
- Students submitted data to the project's database (3)
- Students analyzed or graphed data from the project (e.g. in Excel, or through the project website) (4)
- Students developed their own scientific hypothesis in the context of the project (5)
- Students tested their own scientific hypotheses in the context of the project (6)
- Other: (7) ________________________________________________

Q2.11 Course 1: What were your learning objectives related to the use of that citizen science project in your class? (check all that apply)

- To illustrate specific content covered by the project (10)
- To reinforce a specific aspect of the scientific process (e.g., sampling, graphing, etc.) (11)
- To expose students to authentic scientific research (12)
- To allow students to go through the entire scientific process with an independent question (13)
- To show students the relevance of science to the "real world" (14)
- To get students excited about science (15)
- To introduce students to a means of contributing to science and society after graduation (16)
- To show diverse role models and/or discuss issues of social justice (17)
- Other (18)

Display This Question:

If Q2.11 = Other

Q2.12 Please describe the "other" learning objectives related to the use of that citizen science project you had in your class?

________________________________________________________________

Q2.13 Course 1: Did you use any informal or formal assessments of learning outcomes or self-efficacy related to using citizen science in your class?

- Yes (1)
- No (2)

Skip To: Q2.15 If Q2.13 = No

Q2.14 Course 1: Please describe what you assessed and how you assessed it. Please include any relevant information, including (but not limited to):

·       whether you created your own assessment or used one from the literature

·       the names of any assessments used from the literature

·       whether you assessed students in class or as homework

·       whether the assessment focused on content, specific scientific skills/practices, motivation, self-efficacy, engagement, overall course grades, or something else

·       whether the assessment was a stand alone assignment or embedded in something else (e.g., a class final exam)

________________________________________________________________

Q2.15 Course 1: Did you use any other citizen science projects (in their entirety or even part) in this same undergraduate course?

- Yes (1)
- No (2)

Skip To: Q2.31 If Q2.15 = No

**Q2.16 – Q2.23 are the same as Q2.7 – Q2.15, for a second citizen science project in Course 1*

**Q2.24 – Q2.31 are the same as Q2.7 – Q2.15, for a third citizen science project in Course 1*

End of Block: Course 1

Start of Block: Course 2

Q3.1 Please complete the following questions about this second undergraduate course in which your students have used part, or all, of a citizen science project that is accessible to individuals outside of your course.

**Q3.2 – Q3.15 are the same as Q2.2 – Q2.15, for a first citizen science project in Course 2*

**Q3.16 – Q3.23 are the same as Q2.7 – Q2.15, for a second citizen science project in Course 2*

**Q3.24 – Q3.31 are the same as Q2.7 – Q2.15, for a third citizen science project in Course 2*

End of Block: Course 2

Start of Block: Course 3

Q4.1 Please complete the following questions about this third undergraduate course in which your students have used part, or all, of a citizen science project that is accessible to individuals outside of your course.

**Q4.2 – Q4.15 are the same as Q2.2 – Q2.15, for a first citizen science project in Course 3*

**Q4.16 – Q4.23 are the same as Q2.7 – Q2.15, for a second citizen science project in Course 3*

**Q4.24 – Q4.29 are the same as Q2.7 – Q2.14, for a third citizen science project in Course 3*

End of Block: Course 3

Start of Block: End Block

Q5.1 What do you feel has gone well with your use of citizen science in undergraduate courses?

________________________________________________________________

Q5.2 What challenges or barriers have you faced when incorporating citizen science in undergraduate courses?

________________________________________________________________

Q5.3 What resources do you think would be beneficial to you, or other instructors, to facilitate the use of citizen science in undergraduate courses?

- List of validated evaluation instruments (4)
- Examples of how other instructors use citizen science in undergraduate classes (5)
- Mapping learning objectives to specific citizen science project characteristics (6)
- University-level lesson plans for specific citizen science projects (7)
- Single website compiling existing resources useful for using citizen science in undergraduate courses (8)
- Other (9) ________________________________________________

Q5.4 Please check here if you would be interested in receiving future emails about incorporating citizen science in undergraduate biology or environmental sciences classrooms.

- Yes, I would be interested (1)
- No, thank you (2)

End of Block: End Block

**Supplemental Table 3**. List of citizen science projects reported as being used in the literature review or the instructor survey.

| **Citizen science project** | **Reported use** |
| --- | --- |
| Auburn Squirrel Project | Instructor Survey |
| Audubon Christmas Bird Count:  La Selva Birds Abundance Trends Analysis | Instructor Survey |
| Backyard Bark Beetles | Vitone et al. 2016 |
| Backyard Worlds - Planet 9 (Zooniverse) | Instructor Survey |
| Barcode of Life | Instructor Survey |
| Big Sea Survey | Instructor Survey |
| Biotracker | Bowser et al. 2013 |
| Bird Point Count Database | Instructor Survey |
| BOKUroadkill (now Project Roadkill) | Heigl and Zaller 2014 |
| Budburst | Instructor Survey |
| Budburst | Bowser et al. 2013 |
| Bumble Bee Watch | Instructor Survey |
| CALeDNA | Instructor Survey |
| CamClickr | Voss and Cooper 2010 |
| Capturing Our Coast | Instructor Survey |
| Caterpillars Count | Instructor Survey |
| Celebrate Urban Birds | Instructor Survey |
| Cell Slider | Kridelbaugh 2016 |
| Chicago Wildlife Watch (Zooniverse) | Instructor Survey |
| Chimp & See (Zooniverse) | Instructor Survey |
| Citizens Statewide Lake Assessment Program | Instructor Survey |
| Climate Watch | Mitchell et al. 2017 |
| CoralWatch | Instructor Survey |
| Cyclone Center | Phillips et al. 2018 |
| EarthTrek Gravestone Project | Guertin 2014 |
| eBird | Instructor Survey |
| eBird | Surasinghe and Courter 2012 |
| EMammal | Instructor Survey |
| ESS 220 through citsci.org | Instructor Survey |
| Etch A Cell (Zooniverse) | Instructor Survey |
| Etch A Cell - Powerhouse Hunt (Zooniverse) | Instructor Survey |
| EteRNA | Instructor Survey |
| ETick | Instructor Survey |
| Eye for Diabetes (Zooniverse) | Instructor Survey |
| Eyewire | Instructor Survey |
| Fern Watch | Instructor Survey |
| Reef Environmental Education Foundation Fish Surveys | Instructor Survey |
| Flip the Clinic | Kridelbaugh 2016 |
| Floating Forests VI (Zooniverse) | Instructor Survey |
| Foldit | Instructor Survey |
| FrogWatch | Instructor Survey |
| Galaxy Zoo (Zooniverse) | Instructor Survey |
| Georgia Adopt-A-Stream | Instructor Survey |
| Global Biodiversity Information Facility (GBIF) | Instructor Survey |
| Global Learning and Observations to Benefit the Environment Program (GLOBE) | Instructor Survey |
| Great Sunflower Project | Oberhauser and LeBuhn 2012 |
| iMapInvasives Network | Instructor Survey |
| iNaturalist | Andrews and Ho 2019 |
| iNaturalist | Instructor Survey |
| Insect DNA Barcoding Project | Instructor Survey |
| James River Association - RiverRats Program | Instructor Survey |
| Journey North | Instructor Survey |
| Lost Ladybug Project | Instructor Survey |
| Maine Bumble Bee Atlas | Instructor Survey |
| Maine Butterfly Survey | Instructor Survey |
| Maine Damselfly and Dragonfly Survey | Instructor Survey |
| MakerNurse | Kridelbaugh 2016 |
| Marine Debris Tracker | Instructor Survey |
| Michigan Cooperative Lakes Monitoring Program (MiCorps) | Instructor Survey |
| Michigan Volunteer Stream Monitoring Program (MiCorps) | Instructor Survey |
| Microplants | Von Konrat et al. 2018 |
| Monarch Larva Monitoring Project | Oberhauser and LeBuhn 2012 |
| Monarch Watch | Instructor Survey |
| NanoDoc | Kridelbaugh 2016 |
| National Phenology Network (U.S.A.) | Instructor Survey |
| Nature Atlas | Hardy and Hardy 2018 |
| Nature's Notebook | Instructor Survey |
| Neighbourwoods Raleigh Project | Instructor Survey |
| North American Amphibian Monitoring Program | Cosentino et al. 2014 |
| North American Amphibian Monitoring Program | Instructor Survey |
| Notes from Nature I | Instructor Survey |
| Notes from Nature (Zooniverse) | Kridelbaugh 2016 |
| Oklahoma Blue Thumb | Instructor Survey |
| Old Weather | Instructor Survey |
| Pennsylvania Mammal Atlas | Instructor Survey |
| Phylo DNA Puzzle | Kridelbaugh 2016 |
| Planet 4: Terrains (Zooniverse) | Instructor Survey |
| Play to Cure: Genes in Space | Kridelbaugh 2016 |
| Reverse the Odds | Instructor Survey |
| School of Ants | Vitone et al. 2016 |
| SeagrassSpotter | Instructor Survey |
| Shady Invaders | Instructor Survey |
| Shark Tooth Forensics | Instructor Survey |
| Snapshot Safari (Zooniverse) | Instructor Survey |
| SquirrelMapper | Instructor Survey |
| The Boulder Apple Tree Project | Instructor Survey |
| Urban Wildlife Information Network | Instructor Survey |
| Vernal Pools Cooperative of Virginia | Instructor Survey |
| Virginia Save Our Streams | Instructor Survey |
| Where Is Delftia? | Instructor Survey |
| Where Is Delftia? | Riley et al. 2020 |
| Wildcam Gorongosa (Zooniverse) | Instructor Survey |
| World Water Monitoring Challenge (now EarthEcho Water Challenge) | Instructor Survey |
| World Water Monitoring Challenge (now EarthEcho Water Challenge) | Guertin 2014 |
